# Acclimation of the Resurrection Plant *Haberlea rhodopensis* to Changing Light Conditions

**DOI:** 10.3390/plants13223147

**Published:** 2024-11-08

**Authors:** Katya Georgieva, Gergana Mihailova

**Affiliations:** Institute of Plant Physiology and Genetics, Bulgarian Academy of Sciences, Acad. G. Bonchev Str., Bl. 21, 1113 Sofia, Bulgaria; gmihailova@bio21.bas.bg

**Keywords:** resurrection plants, desiccation, high light intensity, stress markers, photochemical activity, antioxidant enzymes

## Abstract

Resurrection plants present an attractive model for studying the mechanisms of desiccation tolerance. In addition to drought, the presence of light during desiccation is extremely dangerous. In the present study, we investigated the effect of light during the desiccation of shade and sun *Haberlea rhodopensis* from two different habitats by measuring the changes in electrolyte leakage, malondialdehyde and proline content, and photosynthetic and antioxidant activities. Moreover, the plasticity and acclimation ability of plants to changing light intensities were studied by desiccating shade plants under high light and sun plants under low light. The most significant differences between shade and sun plants were observed under moderate dehydration. Regardless of some decline in PSII activity in sun plants, it was much higher compared to shade plants. The lower PSII efficiency in the latter was accompanied by a higher extent of excitation pressure and consequently significant enhancement in non-photochemical quenching, Y(NPQ), and especially in the fraction of energy that is passively dissipated as heat and fluorescence, Y(NO). The activity of antioxidant enzymes remained high during the desiccation of *H. rhodopensis*, being higher in the sun compared to shade plants in an air-dried state. In addition, shade and sun plants showed high acclimation capacity when desiccated at opposite light intensities.

## 1. Introduction

*Haberlea rhodopensis* is one of the rare resurrection plants that can withstand desiccation to an air-dry state and fully recover upon rehydration. It is a tertiary relict and an endemic plant of the Balkans that grows on rock surfaces at elevations ranging from 136 to almost 1600 m above sea level under varying temperatures, light intensities, and humidities [1]. *H. rhodopensis* belongs to the group of homoiochlorophyllous resurrection plants, which retain most of their chlorophyll content during desiccation without destroying the photosynthetic apparatus. This allows for the rapid recovery of dry plants after rehydration, but chlorophyll molecules are a source of reactive oxygen species (ROS). Furthermore, stomata closure during drought stress prevents the use of absorbed light energy to drive CO_2_ fixation, leading to an increase in superoxide anion radical formation at photosystem I (PSI) and singlet oxygen at PSII [2]. *H. rhodopensis* is a shade plant growing at very low light intensities under trees (about 25–30 µmol m^−2^ s^−1^), and it can rarely be found on rocks exposed to full sunlight. Exposure to excessive energy, arising either from high irradiance or as a consequence of drought stress, is detrimental and can cause photoinhibition and significant subcellular damage [3]. It has been shown that when resurrection plants are dried in high light as opposed to low light, there is greater damage and a longer recovery period [4,5]. Thus, studying the effects of light during the desiccation of resurrection plants is important. In our previous studies, we used two approaches to investigate the effect of light during the desiccation of *H. rhodopensis*: shade plants were exposed to different light intensities, or the response of plants growing in understory shaded habitats and in sunny open environments was compared. It was found that *H. rhodopensis* taken from a shaded environment is very sensitive to photoinhibition [6]. Desiccation of plants under an irradiance of 350 µmol m^−2^ s^−1^ induced irreversible changes in the photosynthetic apparatus, and only young leaves recovered after rehydration [7]. To better understand how *H. rhodopensis* sun plants cope with high radiation, we compared their response to desiccation with that of shade plants, and we found both similarities and differences. Sun plants have smaller leaves and tighter, funnel-shaped rosettes with a steep leaf angle [8]. Moreover, the morphology of both ecotypes differed during dehydration. Leaf folding of shade plants was observed when relative water content (RWC) dropped below 50% and leaves shrank parallel to desiccation, regardless of growth irradiance. However, leaf curling was more pronounced in sun plants, their leaves were tightly packed in the desiccated state, and the oldest leaves turned brown, protecting the inner leaves from photoinhibition. Leaf folding and shading of inner leaves decreased the light–chlorophyll interactions and oxidative damage [9,10]. Surfaces exposed to light accumulate “sun screen” pigments, such as anthocyanins and xanthophylls, which reflect photosynthetically active light, mask chlorophyll, and function as antioxidants [11,12,13]. Furthermore, it was discovered that *Craterostigma wilmsii* leaves prevented from folding die when dried in the light but not in the dark [12].

In addition, our studies showed the importance of polyphenols and carbohydrates for the desiccation tolerance of shade and sun *H. rhodopensis* plants [14,15]. The total amount of polyphenols was higher in the well-hydrated sun plants compared to the shade plants, mainly due to their twofold higher content of myconoside and paucifloside. Desiccation to an air-dry state further increases their content, especially in shade plants [14]. Polyphenols have been shown to protect membranes from desiccation-induced damage by integrating into the lipid bilayer and having free radical scavenging capacity [16,17]. Similar to polyphenols, the total amount of soluble carbohydrates increased more in desiccated shade *H. rhodopensis* plants compared to sun plants, reaching a maximum in dry leaves [15]. Sucrose was the most abundant saccharide during desiccation in both ecotypes but also increased to a greater extent in dehydrating shade plants. On the other hand, raffinose content was higher not only in control sun plants than in shade plants, but also during desiccation. The important role of sugars in preserving the membranes under severe water loss by water replacement was reported [18]. Raffinose has been shown to enhance the protective properties of sucrose and contribute to protection against oxidative damage [19,20]. This suggests the important role of raffinose in the high light tolerance of sun plants.

We also found that sun plants have higher rates of CO_2_ assimilation and photochemical activity of PSI and PSII not only in the well-hydrated state but also during dehydration, which is accompanied by reduced susceptibility to photodamage [21,22]. Furthermore, a characteristic feature of sun plants is that the stomata are only visible on the abaxial leaf side, where there is also a greater abundance of non-glandular trichomes [23]. Surprisingly, although sun and shade plants were phenotypically different, only minor differences in chloroplast ultrastructure are observed, such as wider grana in shade compared to sun chloroplasts [8]. The densely packed leaves of sun plants with a perpendicular position to the sunlight probably shade each other more or less, reducing the light intensity at the chloroplast level. In agreement with this, shade and sun plants showed only slight differences in the organization of chlorophyll–protein complexes. Despite the greater abundance of most complexes in sun leaves, the ratios of the main complexes, providing information on modifications in thylakoid organization, were similar in both populations. The main difference was the higher ratio of Lhc monomers to LHCII trimers in sun plants, which could be an acclimation mechanism related to the creation of a quenching state due to LHCII monomerization under excessive light [24]. Indeed, we found that in response to desiccation, sun plants rely more on the protection of the quenching mechanism in the light-harvesting antennae, while shade plants rely more on the re-emission of excitation energy from inactivated PSII reaction centers [21].

In addition to non-photochemical quenching, upregulation of antioxidant systems during desiccation is crucial to minimize photooxidative damage [25]. Maintenance of high antioxidant activity upon desiccation is a distinguishing feature in resurrection plants, but there is considerable variation in changes in non-enzymatic antioxidants and antioxidant enzymes between different resurrection plants in response to desiccation [13].

Thus, resurrection plants present an attractive model for studying the mechanisms of coping with extreme drought without irreversible damage to their tissues. Investigating the desiccation tolerance mechanisms of *H. rhodopensis* growing under low and high radiance under natural conditions contributes to a better understanding of the effect of light during desiccation, which largely endangers plant survival. In addition, this knowledge may help in the development of strategies to improve abiotic stress tolerance in crop species [26,27]. However, studies on the effect of different growth irradiances during the desiccation–rehydration cycles received less attention. In the present study, we first compare the responses of sun and shade *H. rhodopensis* plants from habitats different from those previously investigated to better understand the strategies of this resurrection plant to adapt to high light conditions and survive complete desiccation under high irradiance. Additionally, the plasticity and acclimation ability of plants to changing light intensities were studied by desiccating shade plants under high light (shade HL) and sun plants under low light (sun LL), respectively. Changes in some stress markers, such as electrolyte leakage from leaf tissues, malondialdehyde (MDA) and proline content, and photosynthetic and antioxidant activities, were investigated at different degrees of desiccation as well as after plant rehydration.

## 2. Results

**Figure 1:**
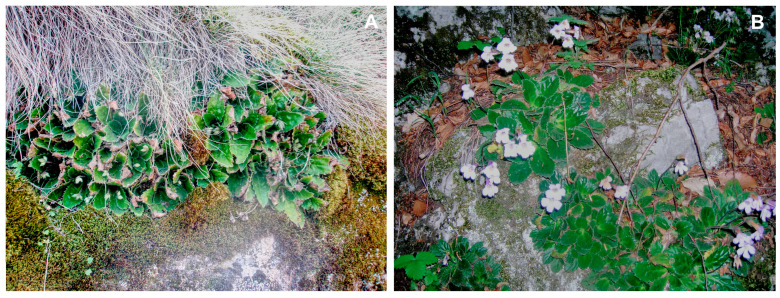
Adult rosettes of well-hydrated *Haberlea rhodopensis* Friv. plants growing on sun-exposed rocks at altitudes of 1150–1200 m in north Rhodopes (sun plants (**A**)) and under deep shade at altitudes of 630–650 m a.s.l. in central Rhodopes (shade plants (**B**)).

Sun plants have small leaves and funnel-shaped rosettes, as previously observed [21]. Although the shade plants have a rosette type similar to that of the previously studied plants, their leaves are smaller and more rounded (Figure 1). Indeed, during desiccation leaf folding and shrinkage occurred, but while the shape of the rosettes of shade plants did not change significantly, the leaves of the sun plants were tightly packed, and the outer leaves turned brown.

### 2.1. Changes in Electrolyte Leakage and Malondialdehyde and Proline Content as Stress Markers

**Figure 2:**
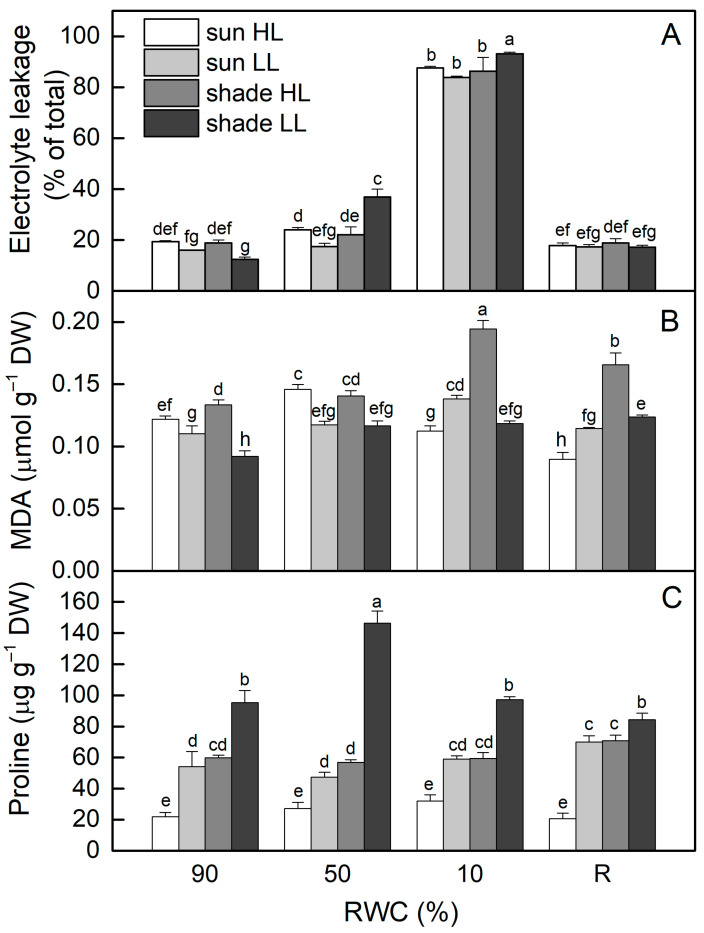
The extent of electrolyte leakage (**A**) and malondialdehyde (MDA (**B**)) and proline content (**C**) in well-hydrated control plants (90% RWC), after moderate desiccation (50% RWC) and in dry leaves (10% RWC), as well as after 4 days of rehydration (R). Four plant groups are used in the experiments: sun plants exposed to high light (sun HL) or low light intensities (sun LL) and shade plants exposed to high light (shade HL) or low light intensities (shade LL). The data represent the mean of *n* = 10; the same letters within a graph indicate no significant differences assessed by Fisher’s LSD test (*p* ≤ 0.05) after performing an ANOVA.

Electrolyte leakage (EL) of well-hydrated (control) shade LL plants was about 35% lower compared to control sun HL plants (*p* ≤ 0.05). Seven days of exposure of control sun plants to LL reduced EL values by 20% (ns), while EL increased after exposure of shade plants to HL, resulting in about a 50% increase in EL (Figure 2A). The most significant two-fold enhancement in EL was observed in shade LL plants under moderate drought stress, whereas EL increased by 24% in sun HL plants, but it was not statistically significant. Indeed, EL increased enormously when RWC dropped to about 10%, regardless of the light intensity. However, it was reversible and close to the control levels after 4 days of rehydration.

The results presented in Figure 2B show that, similar to EL, MDA content was lower in shade LL plants (about 25%) compared to sun HL plants (*p* ≤ 0.05). Treatment of control shade LL plants at high light resulted in a 45% enhancement in MDA, while its content decreased in sun LL plants. Dehydration of sun HL and shade LL plants increased MDA content (*p* ≤ 0.05), but no additional changes were detected in sun LL and shade HL plants. In the dry state (10% RWC), the most significant increase in MDA was observed in desiccated shade HL plants. MDA content remained higher than controls after rehydration of the latter and also in shade LL plants.

The proline content of well-hydrated shade LL plants was much higher (3-fold) compared to that of sun HL plants (Figure 2C). Exposure of control sun plants to low light significantly increased proline content, while exposure of shade plants to high light decreased its content, resulting in similar proline content in them in a well-hydrated state. During the desiccation of all plant groups, significant changes in proline content were observed only in shade LL plants. The highest amount of proline was detected at 50% RWC and then it decreased to the control level in dry *H. rhodopensis* plants.

### 2.2. Photochemical Activity of PSII and Pigment Content

**Figure 3:**
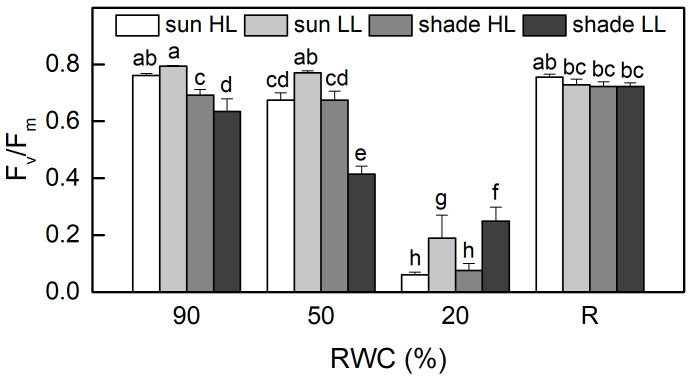
Maximum quantum efficiency of PSII in a dark-adapted state, estimated by the ratio F_v_/F_m_ in well-hydrated control plants (90% RWC), after moderate desiccation (50% RWC) and in dry leaves (10% RWC), as well as after 4 days of rehydration (R). Figure 2 describes plant groups used in the experiments. The data represent the mean of *n* = 10; the same letters within a graph indicate no significant differences assessed by Fisher’s LSD test (*p* ≤ 0.05) after performing an *ANOVA*.

The values of the ratio F_v_/F_m_, as a measure of the maximum efficiency of PSII, were about 20% higher in well-hydrated sun HL plants compared to shade LL plants (*p* ≤ 0.05, Figure 3). When RWC declined to 50%, F_v_/F_m_ was reduced by 10% in sun HL plants, while it decreased by 35% in shade LL plants, suggesting the higher sensitivity of shade plants. However, moderate dehydration of sun LL plants and especially of shade HL plants resulted in an increased quantum efficiency of PSII in a dark-adapted state. It should be noted that the photochemical activity of PSII was measured at 20% RWC, as it was completely inhibited in an air-dry state. The photochemical activity of PSII was significantly inhibited by severe desiccation but fully recovered after 4 days of rehydration.

**Figure 4:**
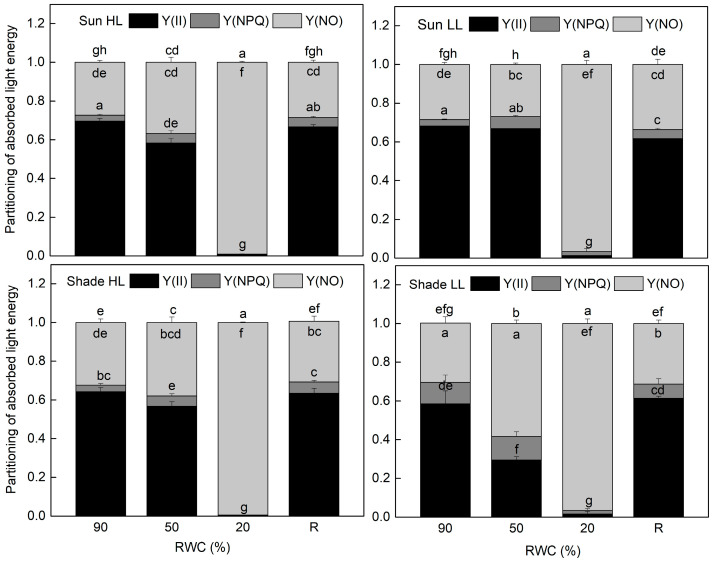
Allocation of absorbed light energy to PSII photochemistry and thus photosynthetic electron transport, Y(II) (**black**), non-photochemical fluorescence quenching, Y(NPQ) (**gray**), and non-regulated thermal energy dissipation, Y(NO) (**light gray**), in well-hydrated control plants (90% RWC), after moderate desiccation (50% RWC) and in dry leaves (10% RWC), as well as after 4 days of rehydration (R). Figure 2 describes plant groups used in the experiments. The data represent the mean of *n* = 10; the same letters within a graph indicate no significant differences assessed by Fisher’s LSD test (*p* ≤ 0.05) after performing an *ANOVA*.

**Figure 5:**
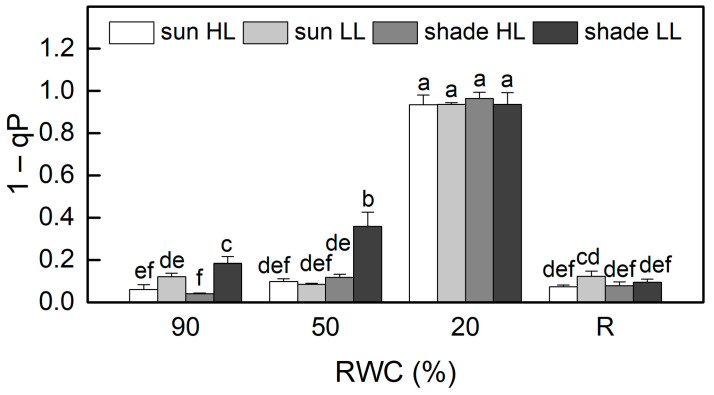
Changes in excitation pressure, estimated by 1 − qP in well-hydrated control plants (90% RWC), after moderate desiccation (50% RWC) and in dry leaves (10% RWC), as well as after 4 days of rehydration (R). Figure 2 describes plant groups used in the experiments. The data represent the mean of *n* = 10; the same letters within a graph indicate no significant differences assessed by Fisher’s LSD test (*p* ≤ 0.05) after performing an ANOVA.

The results of the allocation of absorbed light energy show that the main part of excitation energy in well-hydrated plants is used for photochemistry (Figure 4). Similar to F_v_/F_m_, the quantum efficiency of PSII electron transport during illumination Y(II) was also higher in sun HL compared to shade LL plants. Exposure of control sun plants and especially of the shade plants to low and high light, respectively, increased the quantum yield of PSII. The main differences in the quantum efficiency of PSII between the investigated plant groups were observed under moderate dehydration. The most significant reduction in Y(II) values was determined in shade LL plants, which was accompanied by an almost two-fold increase in excitation pressure, estimated by the values of 1 − qP (Figure 5). Excitation pressure is suggested to be a major prerequisite for inducing efficient dissipation of excess excitation energy, thereby protecting the PSII reaction center from overexcitation. Indeed, the fraction of energy that is passively dissipated as heat and fluorescence, Y(NO), significantly increased in shade LL plants at 50% RWC (Figure 4). Despite some decline (about 15%), the quantum yield of PSII in moderately dehydrated sun HL plants was much higher compared to shade LL plants (*p* ≤ 0.05). In addition, the desiccation of shade HL and sun LL plants to 50% RWC resulted in increased Y(II) values compared to shade LL and sun HL plants. Furthermore, a slight increase in the quantum yield of non-photochemical quenching, Y(NPQ), was observed in all plant groups under moderate desiccation. Except in sun LL plants, some enhancement in Y(NO) was also detected. Severe desiccation of *H. rhodopensis* led to complete inhibition of PSII activity strong enhancement in excitation pressure, and the major part of absorbed light was emitted as Y(NO). But 4 days of rehydration were sufficient to fully restore the effectiveness of PSII.

**Figure 6:**
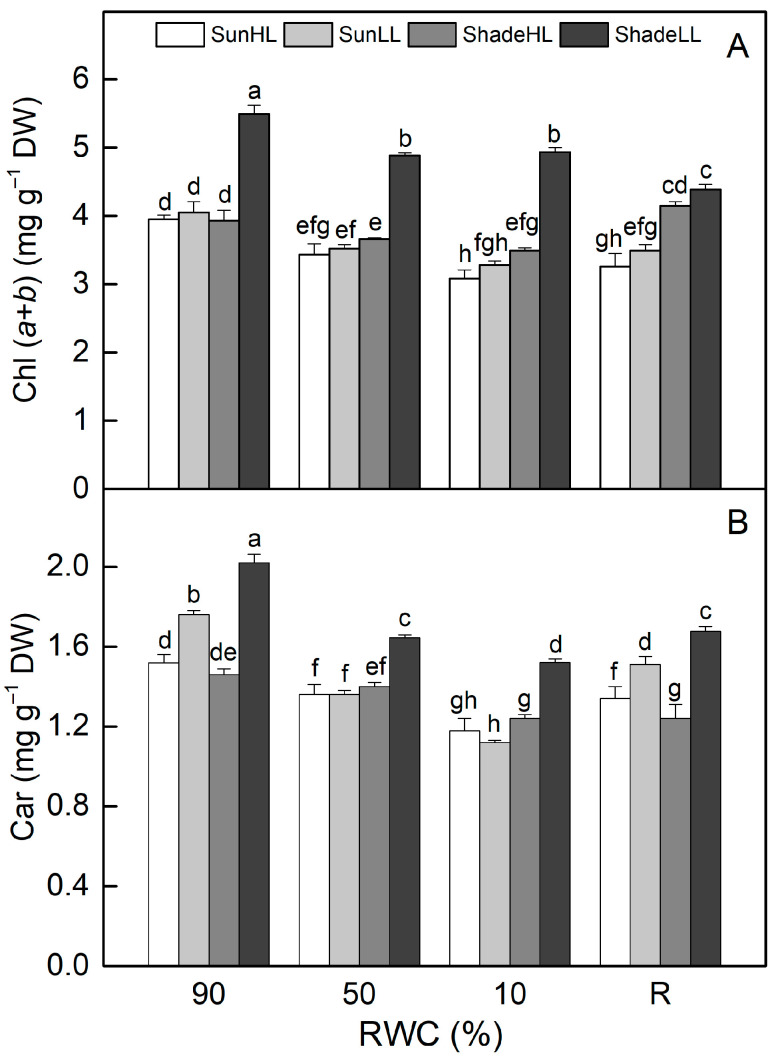
Changes in leaf chlorophyll [Chl (*a* + *b*)] (**A**) and carotenoid [Car] (**B**) content in well-hydrated control plants (90% RWC), after moderate desiccation (50% RWC) and in dry leaves (10% RWC), as well as after 4 days of rehydration (R). Figure 2 describes plant groups used in the experiments. The data represent the mean of *n* = 10; the same letters within a graph indicate no significant differences assessed by Fisher’s LSD test (*p* ≤ 0.05) after performing an ANOVA.

The chlorophyll (Chl) (*a* + *b*) content was higher in shade LL compared to sun HL plants and remained higher during desiccation (*p* ≤ 0.05, Figure 6A). In addition, desiccation to an air-dry state resulted in a higher reduction in Chl content in sun plants. Chl content decreased in well-hydrated shade HL plants and was further reduced by 11% as a result of severe desiccation (*p* ≤ 0.05). The treatment of well-hydrated sun plants at LL slightly increased Chl content, and it was somewhat higher than sun HL plants upon desiccation, but the differences were not statistically significant. Similar changes were observed in the carotenoid content of all plant groups (Figure 6B). The content of photosynthetic pigments was not completely recovered after 4 days of rehydration.

### 2.3. Effect of Light on the Activity of Antioxidant Enzymes During Desiccation

**Figure 7:**
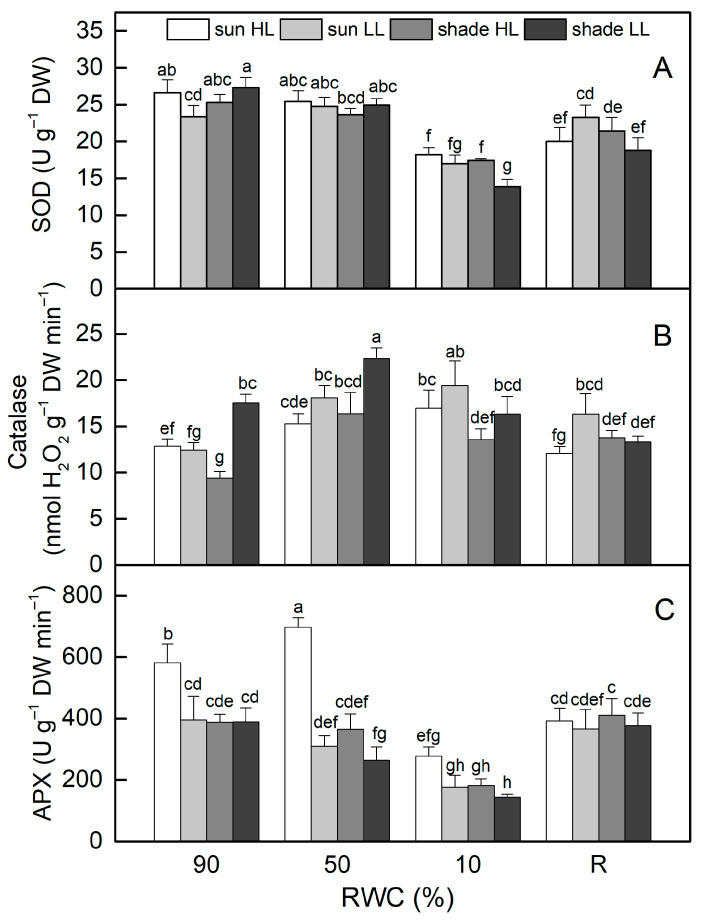
The activity of superoxide dismutase, SOD (**A**), catalase (**B**), and ascorbate peroxidase, APX (**C**), in well-hydrated control plants (90% RWC), after moderate desiccation (50% RWC) and in dry leaves (10% RWC), as well as after 4 days of rehydration (R). Figure 2 describes plant groups used in the experiments. The data represent the mean of *n* = 10; the same letters within a graph indicate no significant differences assessed by Fisher’s LSD test (*p* ≤ 0.05) after performing an ANOVA.

The activity of superoxide dismutase (SOD) was similar in control sun and shade plants (Figure 7A). There were no significant changes in its activity when plants were dehydrated to 50% RWC. Desiccation to an air-dry state resulted in reduced SOD activity by 30% in sun HL plants and more significantly in shade LL plants (by 50%). Despite some enhancement after rehydration, it remained lower than controls in both plant groups. SOD activity slightly declined when well-hydrated sun and shade plants were exposed to LL and HL, respectively. Desiccation of sun LL and shade HL plants to an air-dry state resulted in about 30% reduction in SOD activity, but it should be mentioned that the activity of SOD was higher in shade HL plants than in shade LL plants (*p* ≤ 0.05).

In contrast to SOD, catalase (CAT) activity rose as sun HL plants desiccated, reaching its highest level when plants were completely dry (Figure 7B). Catalase activity was higher in well-hydrated shade LL plants compared to sun HL plants, increased by 30% in the moderately dehydrated state, and was at a control level of 10% RWC. In addition, the desiccation of sun LL plants resulted in a significant enhancement in CAT activity, and it was 50% higher than that of the control in an air-dry state. Exposure of hydrated shade plants to HL reduced CAT activity, but it increased as a result of desiccation. CAT activity remained higher than that of the control after rehydration of both sun LL and shade HL plants.

The highest ascorbate peroxidase (APX) activity in the hydrated state was determined in sun HL plants, while its activity was similar in the other plant groups (Figure 7B). Dehydration to 50% RWC resulted in a 20% increase in APX in sun HL plants, whereas it significantly decreased in shade LL plants. APX activity was reduced by more than 50% in completely dry plants, regardless of the light intensity. After rehydration, their APX activity increased and was similar in all plant groups.

## 3. Discussion

### 3.1. Response of Sun and Shade Plants to Desiccation

In their natural environments, plants usually face several abiotic stressors at once, particularly extreme light and water shortages. In addition to drought, the presence of light during desiccation is extremely dangerous. Thus, in this study, we investigate how plants protect themselves from photodamage during dehydration using sun and shade *H. rhodopensis* plants as models. Moreover, we investigated the acclimation ability and plasticity of this plant to changing light conditions by exposing shade plants to high light and sun plants to low light intensities.

The sun plants were characterized by a higher extent of EL and MDA content, suggesting that they experience higher stress under their natural conditions compared to shade plants. However, a vertical orientation of the leaves of sun plants and their lower chlorophyll content reduce the received light and, together with higher photosynthetic activity, protect them from photoinhibition. On the other hand, by having a horizontal leaf orientation and higher chlorophyll content, shade plants receive light throughout the day under their natural habitats. Their lower photosynthetic activity compared to sun plants was accompanied by higher chlorophyll fluorescence quenching, which is one of the main protective mechanisms for the dissipation of excess excitation energy that cannot be used for photochemistry. In addition, they have higher proline content. Since the photosynthetic apparatus of plants generates ROS even under optimal environmental conditions, an active antioxidant system is important to maintain the physiological activity of plants. We found that the activity of SOD, acting as a first line of defense against ROS by scavenging the toxic superoxide anion, was similar in shade and sun plants. The product of SOD activity, H_2_O_2_, is scavenged by APX and CAT, whose activity was different in shade and sun plants. While the CAT activity was higher in control shade plants, that of APX was higher in sun plants.

The reduction in RWC to 50% led to a more significant enhancement in EL in shade plants compared to sun plants, suggesting the initiation of membrane modifications. A strong increase in EL in dry plants suggests a disturbance of membrane function [28]. However, in contrast to drought-sensitive plants, changes in the integrity of plant cell membranes in *H. rhodopensis* were reversible, and the degree of EL returned back to that of the control after rehydration. In our previous study, we applied a diffusion model to elucidate the reasons for a significant increase in electrolyte leakage from dry leaves [29]. We found that in moderately dehydrated plants, ion efflux was mainly due to leakage from the apoplast, whereas the electrolyte leakage increased sharply in severely desiccated leaves mainly due to ion efflux from the symplast. We propose that the high extent of leakage in air-dried leaves should not be considered as damage, but rather as a reversible remodeling of the cell wall, plasma membrane, and vacuolar system of the cells as a survival strategy.

The most significant differences in photosynthetic activity between shade and sun plants were observed under conditions of moderate dehydration (50% RWC). Regardless, some sun plants showed a decline in PSII activity, and it was much higher compared to shade plants. The lower PSII efficiency in the latter was accompanied by a higher extent of excitation pressure and, consequently, significant enhancement in Y(NPQ) and especially of Y(NO). As previously detected, non-photochemical quenching, NPQ, had a protective effect mainly under moderate dehydration [30]. It was completely inhibited under severe water stress with the inhibition of photosynthetic activity. In contrast, with increasing the degree of desiccation, the values of Y(NO) strongly increased and the main part of excitation energy in the dry state is dissipated passively as heat. According to Genty et al. [31], higher Y(NO) relative to Y(NPQ) is an indication that excess light causes a severe reduction of PSII acceptors and photodamage. Thus, the drought tolerance of the photosynthetic system depends to a larger extent on the plant’s ability to minimize photoinhibition. Indeed, the wilting of leaves under moderate dehydration and leaf rolling and shrinkage below 50% RWC reduce the light absorption. Decreased chlorophyll content, especially in sun plants, also reduces light absorption. Charuvi et al. [32] observed more substantial chlorophyll degradation in the upper layers of dry *Craterostigma pumilum* leaves that are more exposed to light. Maintaining high PSII activity under moderate dehydration due to the activation of alternative electron flows [2] protects plants from photoinhibition. Furthermore, a controlled reversible shutdown of photosynthesis in an air-dry state represents a defense mechanism to minimize oxidative damage [33,34]. Our previous results showed that the inhibition of photosynthesis in a desiccated state was accompanied by a decreased level of the main proteins of both photosystems [35]. In addition, a notable decrease in Lhc proteins was detected, indicating a potential mechanism for ensuring restricted light absorption and preventing excessive excitation and consequent photodamage [36]. Moreover, the rearrangements in the supramolecular organization of PSII and LHCII were observed in dried *C. pumilum*, resulting in their photochemically quenched states, potentially reducing ROS formation at both photosystems [32,37].

Redox homeostasis is further maintained through an efficient ROS-scavenging antioxidant system and the accumulation of osmolytes. Indeed, our results suggest that *H. rhodopensis* plants keep oxidative stress under control, which is evident from the not-so-pronounced changes in MDA content. Under moderate dehydration, SOD activity was close to the control, and catalase activity increased in both shade and sun plants, while APX increased in shade and declined in sun plants. Regardless of the decline in the activity of SOD and APX in dry leaves, it was still rather high, whereas that of catalase reached the highest values in dried sun plants and was at the control level in shade plants. In general, our results showed that the activity of antioxidant enzymes remained high during the desiccation of *H. rhodopensis*, but it was higher in the sun compared to shade plants in an air-dried state. In contrast to drought-sensitive plants, maintaining high antioxidant activity during desiccation is typical for resurrection plants, indicating that these proteins are well protected from denaturation in dry conditions [12,33,38]. Enhanced antioxidant activity contributes to the maintenance of photosynthetic activity under moderate dehydration and prevents stronger damage under desiccation [39]. It was suggested that the high antioxidant activity in the dry state is important for the protection against ROS during the early stages of rehydration and thus for the complete recovery of plants [13]. However, the response of different antioxidant enzymes varies greatly among different resurrection plants or even in the same species depending on the habitat condition [40]. The increased APX activity during drying was reported for many resurrection plants, such as *Craterostigma wilmsii*, *Myrothamnus flabellifolia*, and *Selaginella bryopteris* [12,41]. The important role of the ascorbate–glutathione cycle in overcoming oxidative stress during drying of *H. rhodopensis* was also established [14]. On the other hand, APX activity was close to the control in fully desiccated *Ramonda nathaliae* and decreased in dried leaves of *Sporobolus stapfianus*. The studies of Tan et al. [36] showed that the enzymatic activities of SOD, APX, and catalase were partially induced by the dehydration of *Boea hygrometrica*. In addition, the transcriptome analysis revealed that desiccation upregulated several SOD and APX enzymes [42]. Moreover, it was found that *H. rhodopensis* has at least eight catalase genes, which is more than in most other species with sequenced genomes, suggesting the role of catalases in the desiccation tolerance of *H. rhodopensis* [42,43].

Accumulation of proline was observed as a result of exposure to different environmental stress factors and was used as a stress marker. Proline has been proposed to have a role in macromolecule stabilization, enzyme protection as a compatible solute, and the storage of carbon and nitrogen for utilization in water deficits and other stresses [44]. However, it was considered that the role of proline as a free radical scavenger in overcoming oxidative damage may be more important than in acting as an osmolyte [45]. We observed much higher proline content in hydrated shade plants compared to sun plants, and it increased further in moderately desiccated shade plants. Some enhancement in its content was determined during the desiccation of sun plants, but it was not statistically significant. Different changes in proline content were reported for different resurrection plants. Živković et al. [46] found some dynamics of the proline content during the desiccation of *Ramonda serbica*, but its amount in desiccated leaves was lower than in well-hydrated ones. Our previous studies showed that proline content increased more when the desiccation of *H. rhodopensis* was carried out at high temperatures [47]. Thus, it could be suggested that the changes in proline content depend on the extent of stress treatment.

### 3.2. Shade and Sun H. rhodopensis Plants Showed High Acclimation Capacity When Desiccated at Opposite Light Intensities

Surprisingly, when shade plants were exposed to and desiccated at high light, the values of the studied parameters reached values close to those of sun plants. It was obvious, especially under moderate desiccation, when there were no statistically significant changes in EL, MDA, pigment content, photochemical activity of PSII, and activity of SOD and catalase between sun HL and shade HL plants. Regardless of the enhancement in the APX activity and strong decline in proline content in shade HL plants, their values did not reach that of sun HL plants. Thus, our data showed the plasticity of *H. rhodopensis* from the studied shade habitat to high light. Light intensity is one of the most important factors affecting the photosynthetic efficiency of plants. It could be proposed that exposure of shade plants growing at about 30 µmol m^−2^ s^−1^ to 650 µmol m^−2^ s^−1^, increased photosynthetic activity, decreased the excitation pressure, and, together with high antioxidant activity, contributed to overcoming the photoinhibition. In fact, comparing the response of two soybean varieties with contrasting shade tolerance to high light, Su et al. [48] found a higher capacity for shade-tolerant varieties to adapt to variations in light conditions compared to shade-intolerant varieties. The importance of the upregulation of antioxidant defense systems for the acclimation of the shaded tea plants to high light stress was also suggested [49].

Contrary, the desiccation of sun plants at low light (sun LL) resulted in decreased values of stress markers and increased photosynthetic activity, indicating some protection by decreasing light intensity. During desiccation, the activity of SOD remained high, and that of catalase increased, while APX activity decreased in sun LL plants and was close to that of shade plants. In general, the results of the desiccation of sun and shade plants to low and high light, respectively, showed their adjustment to the contrasting light conditions, which was confirmed by the complete recovery of all plant groups.

## 4. Materials and Methods

### 4.1. Desiccation and Rehydration of Plants

Shade plants were collected from central Rhodopes, where they grow in deep shade under trees at a light intensity of about 25 µmol m^−2^ s^−1^ at an altitude of 630–650 m. Sun plants were collected from northward-facing slopes of sun-exposed limestone rocks in north Rhodopes at altitudes in the range of 1150–1200 m. Plants were collected from their natural habitats together with the layer of soil on which they grew, in order to prevent damage to the plants, and were transferred within a short time to the laboratory and planted in pots.

After acclimation in a climatic chamber FytoScope FS 130 (Photon Systems Instruments, Drásov, Czech Republic) for 7 days, plants were subjected to drought stress by withholding irrigation at 25/18 °C day/night temperatures, 60% relative humidity, a 12 h photoperiod, and a photosynthetically active photon flux density (PPFD) of 25 μmol m^−2^ s^−1^ for shade plants and 600 μmol m^−2^ s^−1^ (approximately the average light intensity in natural habitats) for sun plants, respectively. In addition, sun plants were desiccated to an air-dry state at a low light intensity (LL) of 25 μmol m^−2^ s^−1^, and shade plants were desiccated at a high light intensity (HL) of 600 μmol m^−2^ s^−1^. Thus, we had the following variants: sun LL, sun HL, shade LL, and shade HL. The measurements were conducted on fully expanded mature leaves from well-hydrated (90% relative water content, RWC), moderately hydrated (approximately 50% RWC), and severely dehydrated plants (10–20% RWC), as well as after 4 days of rehydration of the dry plants (R, approximately 90% RWC). Plants were rehydrated in a modified desiccator, where the desiccant at the bottom was replaced by water, providing permanent high humidity by a water pump.

### 4.2. Determination of RWC

The RWC of leaves was determined gravimetrically by weighing them before and after oven-drying at 80 °C to a constant mass and expressed as a percentage of water content in dehydrated tissue compared with water-saturated tissues using the following equation: RWC (%) = (FW − DW)/(TW − DW)× 100, where FW—fresh weight; DW—dry weight; and TW—turgid weight. TW was measured on leaves maintained for 12–16 h at 4 °C in the dark floating on water.

### 4.3. Pigment Content Determination

In total, 100 mg of leaves was homogenized with 10 mL of 80% acetone, and the homogenate was centrifuged at 5000× *g* for 20 min at 4 °C. The Chl *a*, Chl *b*, and total carotenoid contents were determined spectrophotometrically by measuring the absorbance at 663, 645, and 460 nm using Spekol 11 (Carl Zeiss, Jena, Germany). The pigment content was calculated according to the equations of Lichtenthaler [50]. The data were presented on a dry weight basis.

### 4.4. Electrolyte Leakage

Electrolyte leakage from leaf tissues (0.1:10 ratio of leaf tissue to dH_2_O) was measured with a conductivity meter (EC 215, Hanna Instruments, Woonsocket, RI, USA). After 24 h incubation of leaf disks in double-distilled water on an orbital shaker (OS-20, Boeco & CO GmbH, Hamburg, Germany), the conductivity (μS cm^−1^) of the solution was measured. The maximum leakage of the tissue was determined after boiling the leaves for 15 min at 100 °C. The results are expressed as a percentage of the maximum leakage.

### 4.5. Malondialdehyde Content (MDA)

MDA was determined according to Esterbauer and Cheeseman [51]; 250 mg leaves were homogenized at 4 °C in 2.5 mL of 0.1% trichloracetic acid (TCA) and centrifuged at 15,000× *g* for 30 min at 4 °C. The reaction mixture contained 0.5 mL of the supernatant, 0.5 mL of 0.1% TCA, and 1 mL of 0.5% thiobarbituric acid in 20% (*w*/*v*) TCA. This solution was boiled for 30 min at 95 °C in a water bath, and after centrifugation at 4000× *g* for 10 min, the absorbance was read spectrophotometrically (Spekol 11, Carl Zeiss, Jena, Germany) at 532 and 600 nm for the determination of MDA. The data were calculated on a dry weight basis.

### 4.6. Determination of Proline Content

Proline was determined by the method of Bates et al. [52]; 400 mg leaves were homogenized in 6 mL of 3% aqueous sulfosalicylic acid, and the homogenate was centrifuged at 5000× *g* for 20 min at 4 °C. The extract of 2 mL was mixed with 2 mL of acidified ninhydrin (2.5% ninhydrin dissolved into 3:2 (*v*/*v*) glacial acetic acid/orthophosphoric acid mixture) and 2 mL of glacial acetic acid. The reaction mixtures were kept in a water bath at 100 °C for 1 h, and after cooling in ice, 4 mL toluene was added. The chromophore containing toluene was separated, and the absorbance was read spectrophotometrically at 520 nm (Spekol 11, Carl Zeiss, Jena, Germany). The amount of proline was calculated using a standard curve and expressed on a dry weight basis.

### 4.7. Chlorophyll a Fluorescence Induction

Chl *a* fluorescence induction was measured with a portable fluorometer *PAM-2500* (Heinz Walz GmbH, Effeltrich, Germany). The leaves were dark-adapted for 15 min and PAR of 90 μmol (photon) m^−2^ s^−1^ was used for the measurements. All of the basic parameters used were given by *PamWin-3* software (version 3.05c, Heinz Walz GmbH, Effeltrich, Germany). The maximum efficiency of PSII photochemistry was calculated as F_v_/F_m_ immediately after the predarkening period. The actual efficiency of PSII electron transport during illumination was estimated at a steady state as Y(II) = (F_m_′ − F_s_)/F_m_′ [53]. The quantum yield of light-induced non-photochemical fluorescence quenching was calculated as (Y)NPQ = (F_m_/F_m_′) – 1, and the quantum yield of non-regulated heat dissipation and fluorescence emission was calculated as Y(NO) = F_s_/F_m_. All three yield parameters sum up to 1: Y(II) + Y(NPQ) + Y(NO) = 1. The excitation pressure of PSII, which gives an approximate measure of the reduction state of the first electron acceptor Q_A_ of PSII, was calculated as 1 − qP, as qP is determined by the equation qP = (F_m_′ − F_s_)/(F_m_′ − F_0_) [54]. Ten leaves from every plant species were marked and measured throughout the experiment.

### 4.8. Enzyme Activities

CAT extraction and activity: Leaf material was extracted with 50 mM K-phosphate buffer, pH 7.0, containing 0.1 mM EDTA and 1% PVP. The homogenate was centrifuged at 4 °C and 15,000× *g* for 30 min. Catalase activity was measured according to Brennan and Frenkel [55] by the decline in absorbance at 240 nm by decomposition of H_2_O_2_ at 25 °C.

APX extraction and activity: Leaf material was extracted with 50 mM K-phosphate buffer, pH 7.0, containing 1.0 mM EDTA, 0.1% (*w*/*v*) Triton X-100, and 5 mM ascorbate (added immediately before extraction). After the extraction, the homogenate was centrifuged at 4 °C and 15,000× *g* for 30 min. Ascorbate peroxidase activity was assayed spectrophotometrically by monitoring the rate of hydrogen peroxide-dependent ascorbate oxidation at 290 nm as described by Nakano and Asada [56].

SOD extraction and activity: Leaf material was extracted with 50 mM K-phosphate buffer, pH 7.8, containing 1.0 mM EDTA and 0.1% (*w*/*v*) Triton X-100. After the extraction, the homogenate was centrifuged at 4 °C and 15,000× *g* for 30 min. Superoxide dismutase activity was measured according to Beyer and Fridovich [57]. SOD was assayed by monitoring the superoxide radical-induced nitro blue tetrazolium reduction at 560 nm.

### 4.9. Statistics

Leaves for biochemical analysis were sampled from five different tufts at each time point. For Chl *a* fluorescence induction, the same ten leaves were measured during the experiments. A comparison of means was made by the Fisher least significant difference (LSD) test at *p* ≤ 0.05 following an ANOVA. A statistical software package (StatGraphics Plus, version 5.1 for Windows, The Plains, VA, USA) was used.

## 5. Conclusions

Drought tolerance depends to a great extent on the plant’s ability to minimize photoinhibition. Leaf wilting under moderate dehydration and leaf rolling and shrinkage below 50% RWC, together with decreased chlorophyll content, reduce light absorption. In fact, the most significant differences between shade and sun plants were observed under moderate dehydration. Changes in the content of stress markers suggest that shade plants experience higher stress under these conditions. Regardless of some decline, the PSII activity remained much higher in sun plants compared to shade plants. The lower PSII efficiency in the latter was accompanied by a higher extent of excitation pressure and significant enhancement in non-photochemical quenching, Y(NPQ), and especially in the fraction of energy that is passively dissipated as heat and fluorescence, Y(NO). NPQ had the protective effect mainly under moderate dehydration, while it was completely inhibited under severe water loss. Inhibition of photosynthesis and increased thermal energy dissipation under drought stress is also common for drought-sensitive plants. However, unlike the latter, resurrection plants are able to maintain the integrity of the membranes and the photosynthetic apparatus, reversibly shut down photosynthesis, and maintain high antioxidant activity in an air-dry state, which minimizes the oxidative damage and allows plants to recover faster after rehydration. In addition, the results on the desiccation of sun and shade plants to low and high light, respectively, showed their adjustment to the contrasting light conditions, which was confirmed by the complete recovery of all plant groups.

## Data Availability

All datasets are contained within the article.
